# TLR7 agonist, N6-LS and PGT121 delayed viral rebound in SHIV-infected macaques after antiretroviral therapy interruption

**DOI:** 10.1371/journal.ppat.1009339

**Published:** 2021-02-18

**Authors:** Denise C. Hsu, Alexandra Schuetz, Rawiwan Imerbsin, Decha Silsorn, Amarendra Pegu, Dutsadee Inthawong, Jumpol Sopanaporn, Pornsuk Visudhiphan, Weerawan Chuenarom, Boot Keawboon, Wei Shi, Merlin L. Robb, John R. Mascola, Romas Geleziunas, Richard A. Koup, Dan H. Barouch, Nelson L. Michael, Sandhya Vasan

**Affiliations:** 1 Armed Forces Research Institute of Medical Sciences, Bangkok, Thailand; 2 U.S. Military HIV Research Program, Walter Reed Army Institute of Research, Silver Spring, Maryland, United States of America; 3 Henry M. Jackson Foundation for the Advancement of Military Medicine, Bethesda, Maryland, United States of America; 4 Vaccine Research Center, National Institute of Allergy and Infectious Diseases, National Institutes of Health, Bethesda, Maryland, United States of America; 5 Gilead Sciences, Inc, Foster City, California, United States of America; 6 Beth Israel Deaconess Medical Center, Boston, Massachusetts, United States of America; 7 Center for Infectious Diseases Research, Walter Reed Army Institute of Research, Silver Spring, Maryland, United States of America; Institut Cochin, INSERM U1016, FRANCE

## Abstract

Toll-like receptor 7 (TLR7) agonist and PGT121 (broadly neutralizing antibody, bnAb) administration previously delayed viral rebound and induced SHIV remission. We evaluated the impact of GS-986 (TLR7 agonist) and dual bnAbs on viral rebound after antiretroviral therapy (ART) interruption. Rhesus macaques inoculated with SHIV-1157ipd3N4 were initiated on daily suppressive ART from Day 14 post SHIV inoculation. Active arm animals (n = 8) received GS-986, N6-LS and PGT121 after plasma viral suppression, starting from week 14. GS-986 induced immune activation and SHIV-specific T cell responses but not viral expression in all the active arm animals. After ART interruption, median time to viral rebound was 6 weeks in the active and 3 weeks in the control arm (p = 0.024). In this animal model, the administration of the combination of GS-986 and dual bnAbs was associated with a modest delay in viral rebound. This strategy should be further evaluated to better understand the underlying mechanisms for the induction of virus-specific immune responses and delay in viral rebound.

## Author summary

We evaluated the impact of TLR7 agonist (GS-986) and two broadly neutralizing antibodies (bnAbs) targeting different regions of the HIV envelope (CD4 binding site by N6-LS and V3 glycan by PGT121) in delaying viral rebound during ART interruption in rhesus macaques that were initiated on viral suppressive antiretroviral therapy (ART) 14 days post SHIV-1157ipd3N4 infection. We found that the combination of TLR7 agonist and dual bnAbs delayed viral rebound after ART interruption by 2-fold (from 3 wks in the control arm to 6 wks in the active arm, p = 0.024). This encouraging result independently validated prior findings of delay in viral rebound with TLR7 agonist and a single bnAb (PGT121) by Borducchi et al, Nature, 2018. Importantly, findings were in concurrence despite the performance of the study by an independent research group, in a different macaque colony, with a different strain of SHIV. Moreover, this study intentionally deferred ART initiation by a week, i.e. on day 14 post inoculation to mirror what is logistically feasible in acute HIV infection. Thus, data from this study may potentially more closely reflect the impact of the combination of TLR7 agonist and dual bnAbs on viral rebound in HIV-infected individuals.

## Introduction

The HIV reservoir, consisting of cells that harbor latent HIV-1 proviruses, is a major barrier to HIV remission [1,2]. The kick and kill strategy, where latently infected cells are stimulated to induce viral reactivation that can then be eliminated by immune responses, has been proposed as a potential strategy to achieve HIV remission [3,4]. Broadly neutralizing antibodies (bnAb) have demonstrated anti-viral activity in viremic individuals and delayed viral rebound when administered during analytical treatment interruption [5]. Recently, in the study by Borducchi et al., administration of the Toll-like receptor 7 (TLR7) agonist vesatolimod (also known as GS-9620), with the V3 glycan-dependent bnAb PGT121 during antiretroviral therapy (ART) delayed viral rebound following ART interruption in simian–human immunodeficiency virus (SHIV)-SF162P3-infected rhesus macaques [6]. Furthermore, 5/11 animals did not experience viral rebound after ART interruption. Adoptive transfer studies and CD8-depletion studies also did not reveal virus in these animals. These data suggest that the combination of innate immune stimulation with bnAb administration can target and eliminate the viral reservoir. BnAbs used in combination have shown greater antiviral activity than individual bnAbs in SHIV-infected macaques [7] and humans [8]. Thus, we evaluated the impact of the combination of TLR7 agonist GS-986, a very close analog of vesatolimod, and two bnAbs targeting different regions of the HIV envelope- CD4 binding site by N6-LS [9] and V3 glycan by PGT121 [10] in delaying viral rebound during ART interruption in rhesus macaques that were initiated on viral suppressive ART 14 days post SHIV-1157ipd3N4 infection.

## Results

**Fig 1:**
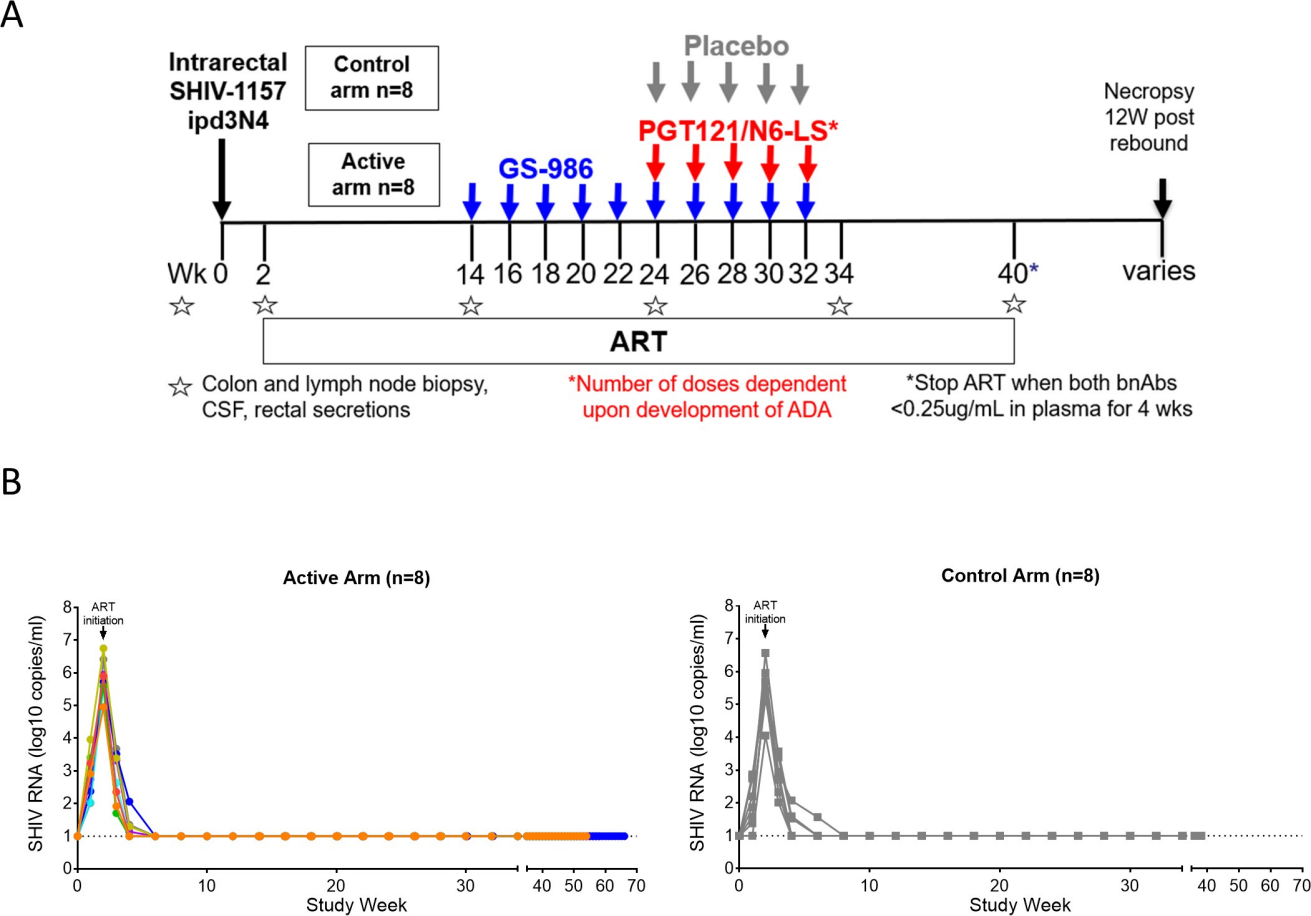
Study schema. **(A)** Male rhesus macaques (n = 16) were inoculated with SHIV-1157ipd3N4 intrarectally at wk 0. ART was initiated on day 14. Animals in the active arm (n = 8) received GS-986 every 2 wks from wk 14 and intravenous N6-LS and PGT121 every 2 wks from wk 24 to 32, unless anti-drug antibodies (ADA) developed. ART was ceased 4 wks after plasma levels of N6-LS and PGT121 were <0.25 ug/mL. Animals in the control arm (n = 8) received normal saline every 2 wks from wk 24 to 32 and ART was ceased at wk 40. (B) Plasma SHIV RNA levels of animals in the active and control arms from the time of inoculation to immediately before ART interruption are shown. Data from animals in the active arm are displayed in color with each color corresponding to individual animals identified in Fig 4A. The x axis has been segmented to allow clearer visualization of viral dynamics during the first 34 wks post infection, when interventions were administered. Dotted line represents limit of quantification (LOQ) of SHIV RNA assay (10 copies/mL).

Sixteen male rhesus macaques (negative for protective MHC-alleles Mamu A-01, B-08 and B-17) were infected by intrarectal inoculation of SHIV-1157ipd3N4. Median day 14 (pre-ART) SHIV RNA load in plasma was 5.70 (interquartile range, IQR 5.37–5.95) log_10_copies/mL and was not different between the active vs control arms (median 5.82, IQR 5.59–6.33 vs median 5.58 IQR 5.23–5.91 log_10_copies/mL, p = 0.33). Animals were initiated on daily ART (PMPA 20 mg/kg, emtricitabine 40 mg/kg and dolutegravir 2.5 mg/kg subcutaneously) on Day 14 (Fig 1A). SHIV RNA became undetectable in the majority of animals by week (wk) 6 and in all animals by wk 8, then remained undetectable until ART interruption (Fig 1B).

### GS-986 administration was associated with increases in immune activation

**Fig 2:**
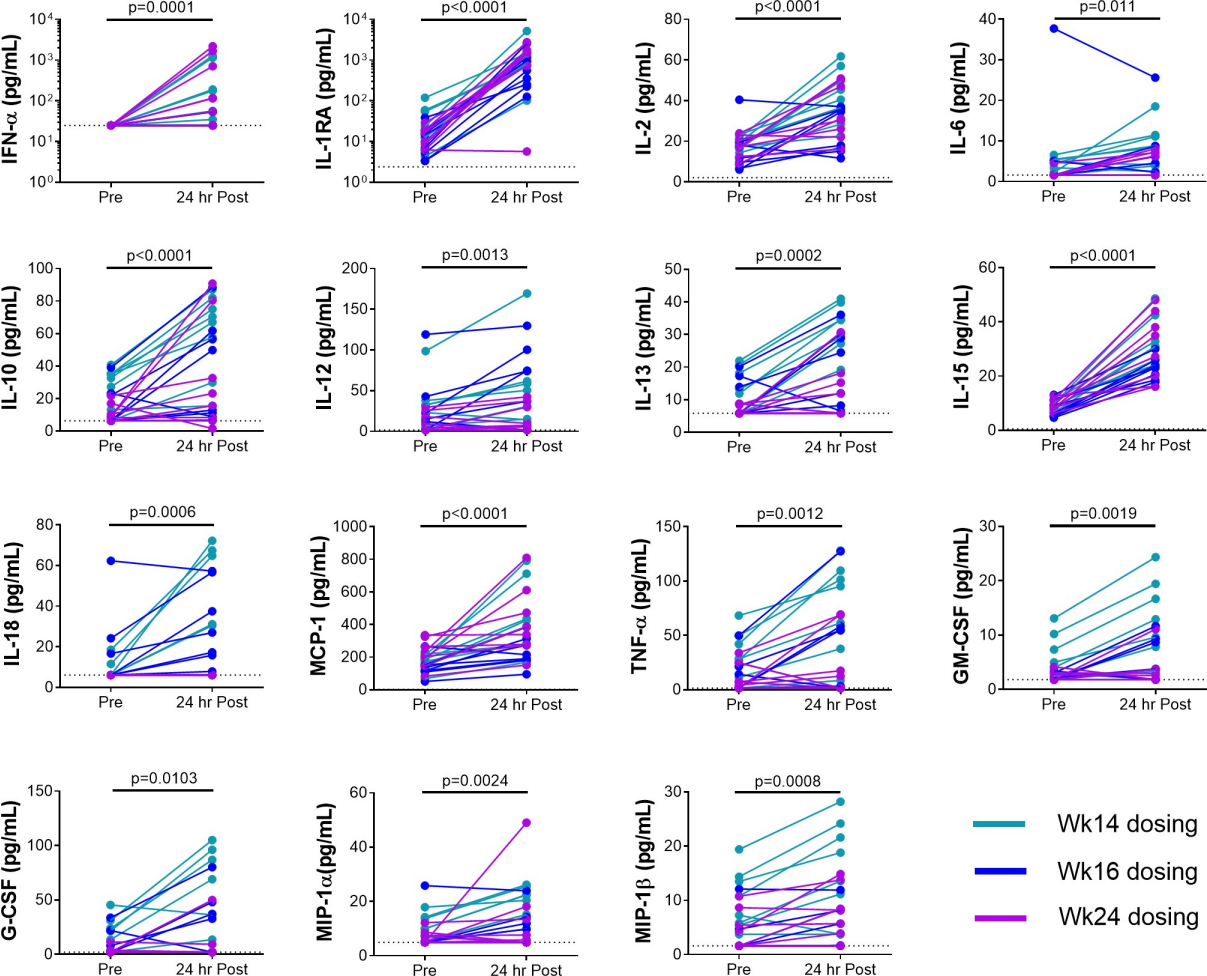
Plasma levels of soluble biomarkers of immune activation pre and 24 hrs post GS-986 administration. Administration of GS-986 was associated with increases in plasma levels of interferon (IFN)-α, Interleukin (IL)-1 receptor antagonist (RA), IL-2, IL-6, IL-10, IL-12, IL-13, IL-15, IL-18, monocyte chemoattractant protein (MCP)-1, tumor necrosis factor (TNF), granulocyte-macrophage colony stimulating factor (GM-CSF), G-CSF, macrophage inflammatory protein (MIP)-1α and MIP1β at 24 hrs post dosing. Data from three dosings (wks 14, 16 and 24) are shown. Dotted lines indicate limit of detection of respective markers. P values were derived using Wilcoxon matched-pairs signed rank test.

**Fig 3:**
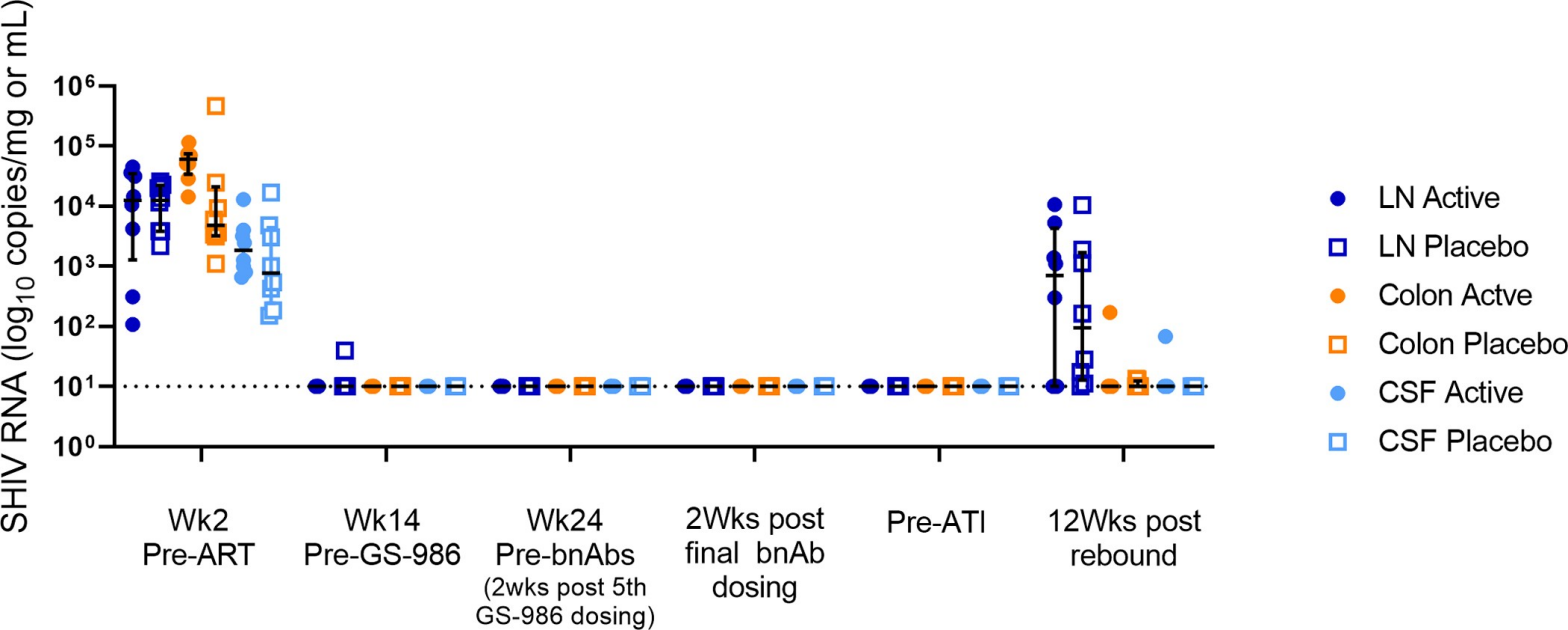
Tissue SHIV RNA levels. Lymph node (blue), colon (orange) and cerebral spinal fluid (CSF, pale blue) SHIV RNA levels were high in the active (filled circle) and control (open square) animals at wk 2 post inoculation. SHIV RNA levels became undetectable in the tissues in all but 1 animal by wk 14 and remained undetectable in all animals whilst on ART. Lines and error bars represent median and interquartile range (IQR). Dotted lines represent LOQ of SHIV RNA assay, 10 copies/mg for lymph node and colon tissues and 10 copies/mL for CSF.

Animals in the active arm (n = 8) received GS-986 (0.1 mg/kg via oral gavage) every 2 wks from wk 14 to 32 (Fig 1A). Administration of GS-986 was associated with increases in plasma levels of interferon (IFN)-α, Interleukin (IL)-1 receptor antagonist (RA), IL-2, IL-6, IL-10, IL-12, IL-13, IL-15, IL-18, monocyte chemoattractant protein (MCP)-1, tumor necrosis factor (TNF), granulocyte-macrophage colony stimulating factor (GM-CSF), G-CSF, macrophage inflammatory protein (MIP)-1α and MIP-1β 24 hours (hrs) post dosing (Fig 2). Plasma SHIV RNA load was monitored 24 hrs after each GS-986 administration and no viral blips were detected. Furthermore, SHIV RNA was also not detected in samples from lymph node, colon and CSF at wk 24 (2 wks after the 5^th^ dose of GS-986) as well as 2 wks after the final administration of GS-986 and bnAbs, suggesting no persistent induction of viral expression in the tissues (Fig 3).

### Plasma and tissue levels of N6-LS and PGT121

**Fig 4:**
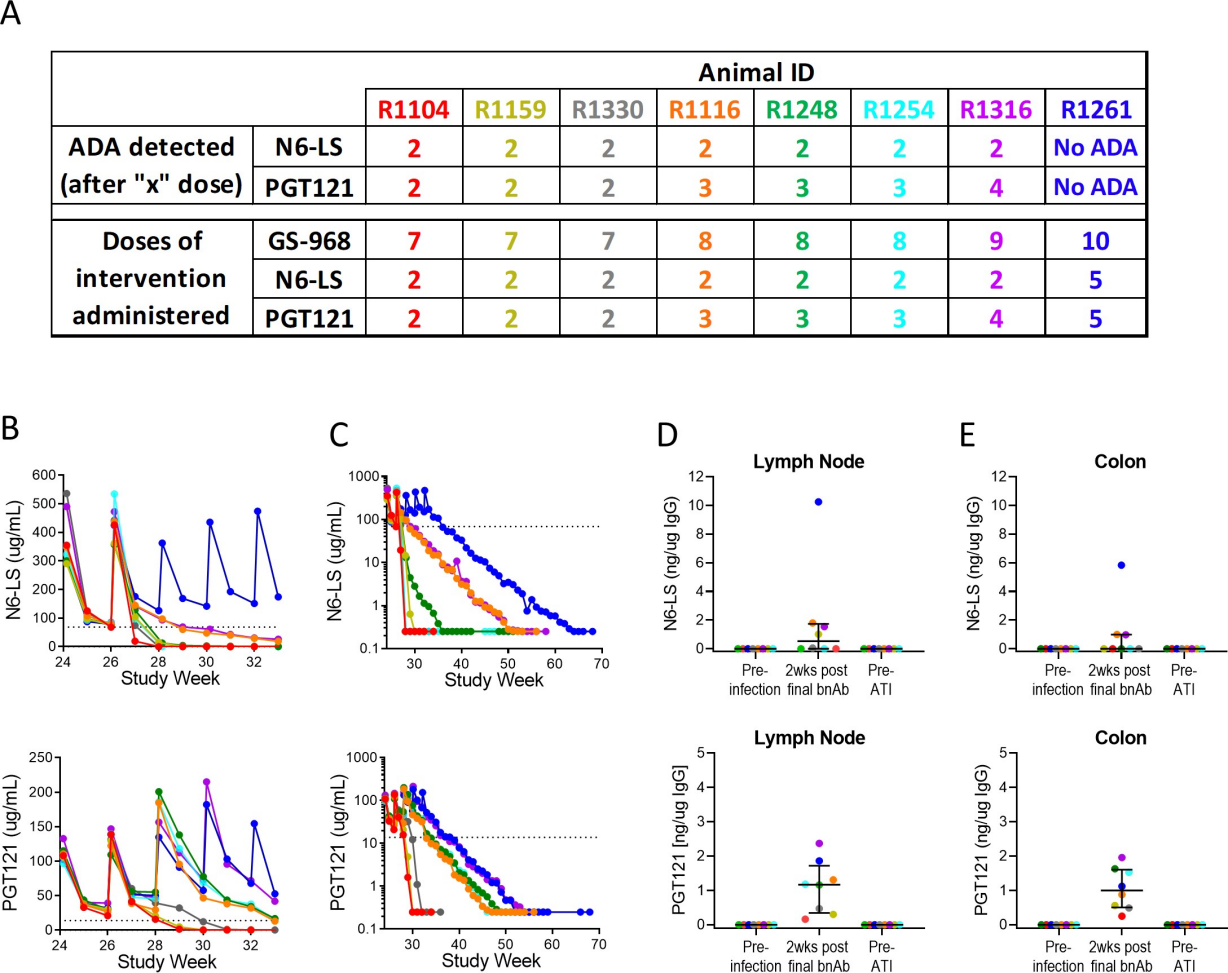
Plasma and tissue levels of N6-LS and PGT121. (A) The number of doses of GS-986, N6-LS and PGT121 administered to each animal varied as no further broadly neutralizing antibody (bnAb) was administered after the detection of anti-drug-antibody (ADA) to the respective bnAb. (B) Peak and trough plasma N6-LS and PGT121 levels during wks 24–33 are shown for each individual animal. Dotted lines represent 100x predicted IC80 levels for SHIV-1157ipd3N4 (0.689 ug/mL for N6-LS and 0.139 ug/mL for PGT121). (C) Plasma levels of both bnAbs were monitored weekly and ART was interrupted when levels for both bnAbs became <0.25 ug/mL. At the time of ART interruption (ATI), N6-LS and PGT121 were both undetectable in (D) lymph node or (E) colon. Lines and error bars represent median and interquartile range (IQR).

Animals in the active arm (n = 8) received intravenous N6-LS (30 mg/kg) and PGT121 (10 mg/kg) every 2 wks from wks 24 to 32, unless anti-drug antibodies (ADA) was detected (Fig 1A). Due to the development of ADA, animals received 7–10 doses of GS-986, 2–5 doses of PGT121 and 2–5 doses of N6-LS (Fig 4A). Only 1 animal (R1261) received all doses of interventions as scheduled.

Plasma N6-LS and PGT121 levels were monitored weekly. Median (range) peak plasma levels (24 hrs after the 1^st^ and 2^nd^ dose) were 335.58 (290.90–536.37) and 431.81 (356.92–534.54) ug/mL for N6-LS and 109.36 (96.86–132.86) and 135.19 (109.45–146.90) ug/mL for PGT121. Median (range) trough plasma levels (2 wks after the 1^st^ and 2^nd^ dose) were 77.95 (68.16–85.99) and 13.77 (0.25–126.44) ug/mL for N6-LS and 29.93 (20.95–39.20) and 42.53 (15.93–55.25) ug/mL for PGT121 (Fig 4B). The rate of decline in plasma N6-LS and PGT121 levels varied between the animals (Fig 4C). The median time from the last administration of bnAb to plasma level <0.25 ug/mL was 7 wks (range 2–32) for N6-LS and 18.5 wks (range 4–24) for PGT121. ART was interrupted 4 wks after plasma levels of both N6-LS and PGT121 were <0.25 ug/mL. Immediately prior to ART interruption, N6-LS and PGT121 were also not detectable in samples from lymph node (Fig 4D) and colon (Fig 4E).

### Impact of the combination of GS-986, N6-LS and PGT121 on SHIV-specific T cell responses

**Fig 5:**
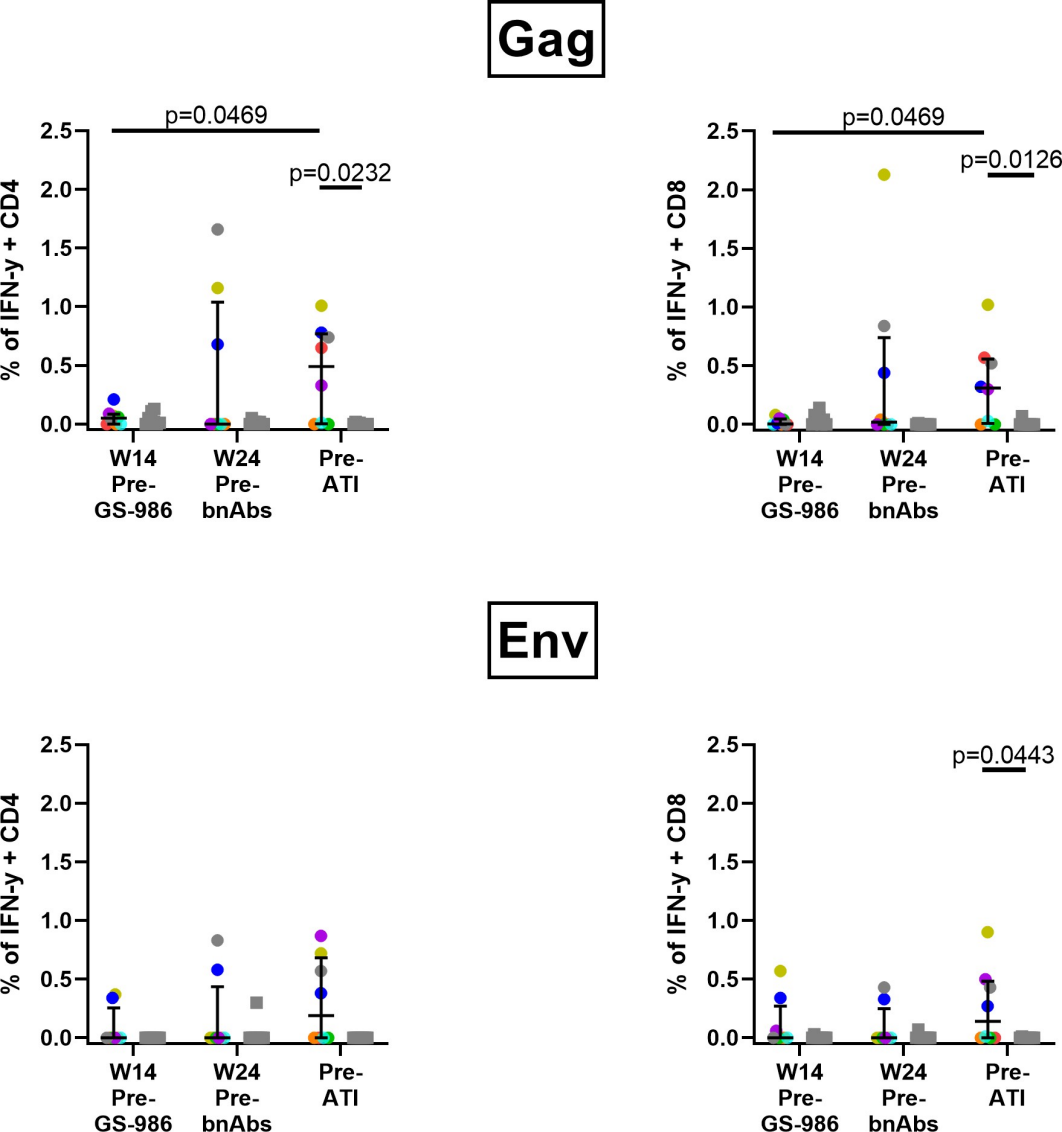
The combination of GS-986, N6-LS and PGT121 induced SHIV-specific T cell responses. The percentage of IFNγ+ CD4 and CD8 T cells after stimulation with SIVmac239 Gag and HIV-1 Consensus C Env peptides are shown for wk 14 (pre-GS-986), wk 24 (2wks after the 5^th^ dose of GS-986, before bnAb administration) and immediately before ART interruption (pre-ATI). Data from animals in the active arm are displayed in color with each color corresponding to individual animals identified in Fig 4A. Data from animals in the control arm are displayed as gray squares.

Interestingly, we observed an induction in SHIV-specific responses in some animals after GS-986 administration. By the time of ART interruption, IFNγ+ Gag-specific CD4 and CD8 T cells in the active arm were significantly higher when compared to wk14 (Pre GS-986, p = 0.0469) as well as to controls (p = 0.0232, p = 0.0126 respectively, Fig 5). A similar trend was observed for IFNγ+ Env-specific CD4 and CD8 T cells (Fig 5). These data suggest that the combination of GS-986, and dual bnAbs increased SHIV-specific T cell responses.

### Impact of the combination of GS-986, N6-LS and PGT121 on Viral DNA levels

**Fig 6:**
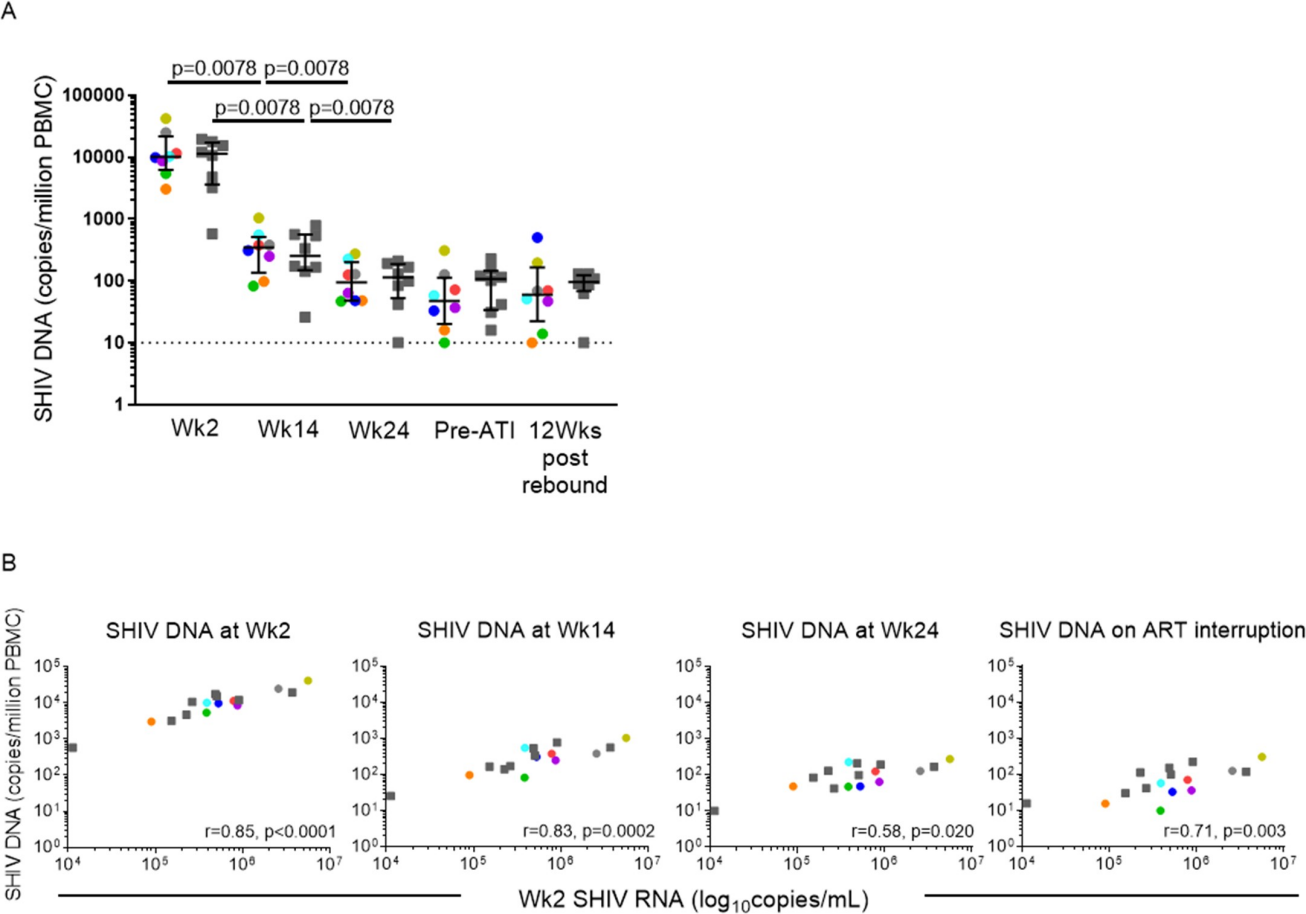
Impact of GS-986 and dual bnAbs on viral DNA levels. (A) Cell-associated viral DNA in PBMC was measured at wks 2 (pre-ART), 14 (pre-GS-986), 24 (2 wks after the 5^th^ dose of GS-986, before bnAb administration), immediately before ART interruption (pre-ATI) and at 12 wks post rebound. Data from animals in the active arm are displayed in color with each color corresponding to individual animals identified in Fig 4A. Data from animals in the control arm are displayed as gray squares. Dotted line represents LOQ of viral DNA assay. (B) Strong correlations were seen between SHIV RNA levels at wk 2 (pre-ART) with SHIV DNA levels at wk 2, wk 14, wk 24 and at ART interruption.

Median SHIV DNA at wk 2 was 10536 (IQR 4930–17275) copies/million PBMC. SHIV DNA levels reduced significantly after ART initiation, to median 323 (IQR 148–550) copies/million PBMC, p<0.0001 at wk 14 and continued to reduce between wks 14 and 24 in both arms. SHIV DNA levels were not significantly different between the arms at all time points measured (Fig 6A). Strong correlations were seen between SHIV RNA levels at wk 2 (pre-ART) with SHIV DNA levels at wk 2 (spearman r 0.85, p<0.0001), wk 14 (spearman r 0.83, p = 0.0002), wk 24 (spearman r 0.58, p = 0.020) and at ART interruption (spearman r 0.71, p = 0.003), suggesting that the level of peak viremia is correlated with the size of the reservoir before and during ART (Fig 6B).

### Delay in viral rebound following ART interruption

**Fig 7:**
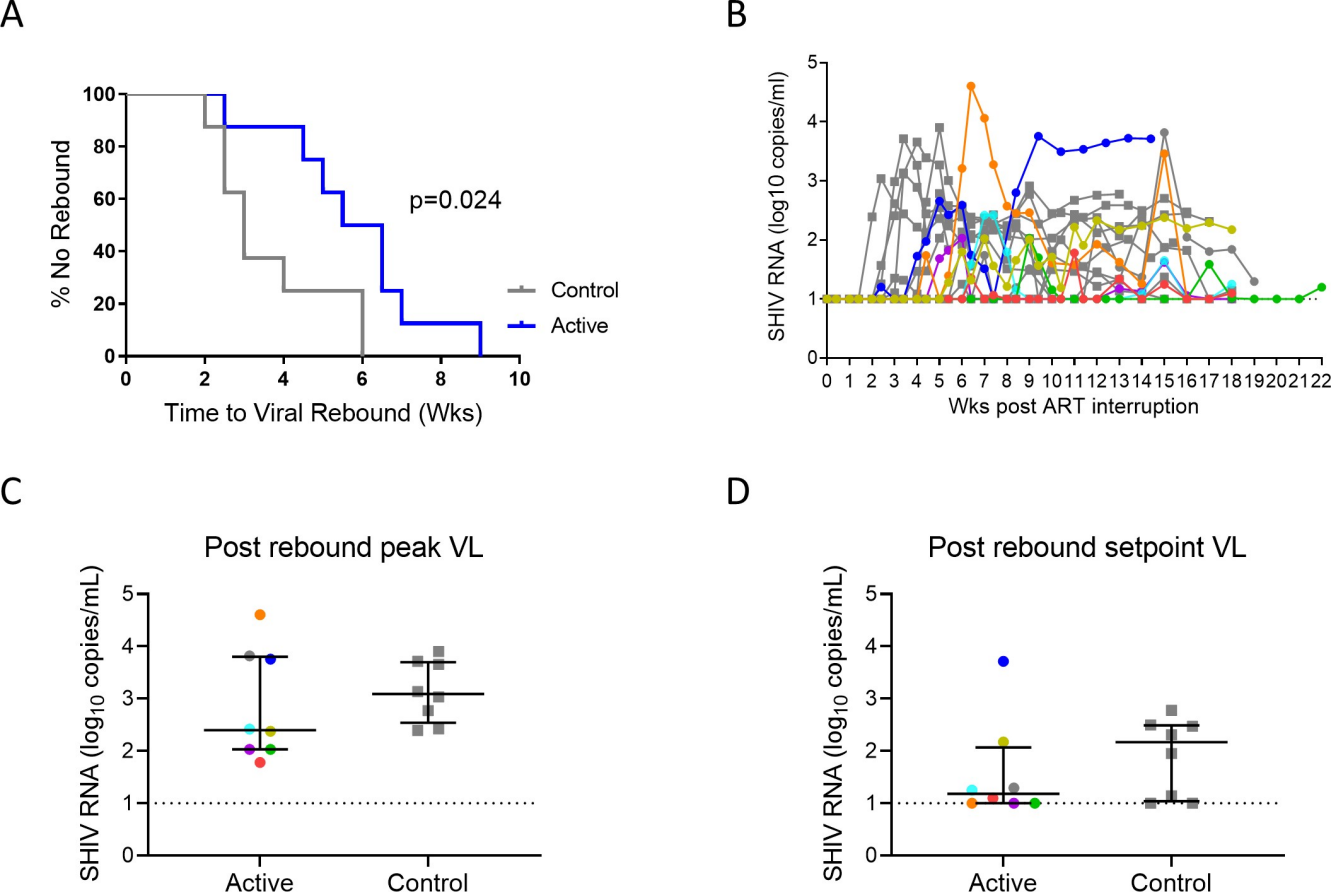
Impact of GS-986 and dual bnAbs on viral rebound after ART interruption. (A) The median time to viral rebound was 3 wks in the control arm (grey) vs 6 wks in the active arm (blue), p = 0.024. (B) Rebound trajectories for individual animals are shown. No differences were seen for (C) post-rebound plasma peak viral load or (D) viral setpoint between arms. Data from animals in the active arm are displayed in color with each color corresponding to individual animals identified in Fig 4A. Data from animals in the control arm are displayed as gray squares. Lines and error bars represent median and interquartile range. Dotted lines represent LOQ of SHIV RNA assay (10 copies/mL).

ART was ceased 4 wks after plasma levels of both N6-LS and PGT121 were <0.25 ug/mL, ranging from study wk 34–68 in the active animals and at wk 40 in control animals (S1A Fig). The median (IQR) duration of viral suppression (SHIV RNA ≤ 10 copies/mL) prior to ART interruption was 48 (30–52) and 36 (34–36) wks in active vs control animals (p = 0.43, S1B Fig). All animals experienced plasma viral rebound after ART interruption. The median (IQR) time to viral rebound was 3 (2.5–5.5) wks in the control arm and 6 (4.6–6.9) wks in the active arm, p = 0.024 (Fig 7A and 7B). There were no differences in post-rebound peak (Fig 7C) viremia between arms. Though more animals in the active arm had lower post-rebound set-point viral load, this did not achieve statistical significance (Fig 7D). There were no correlations between time to rebound with SHIV RNA at wk 2 (S1C Fig), SHIV DNA at ART interruption (S1D Fig) in either arms individually or combined. There was also no correlation between time to rebound and the number of doses of GS-986 and bnAbs administered (S1E and S1F Fig). The animal that received all interventions as scheduled did not experience a longer time to rebound.

### No detectable adverse effects of GS-986, N6-LS and PGT121 in the central nervous system (CNS)

**Fig 8:**
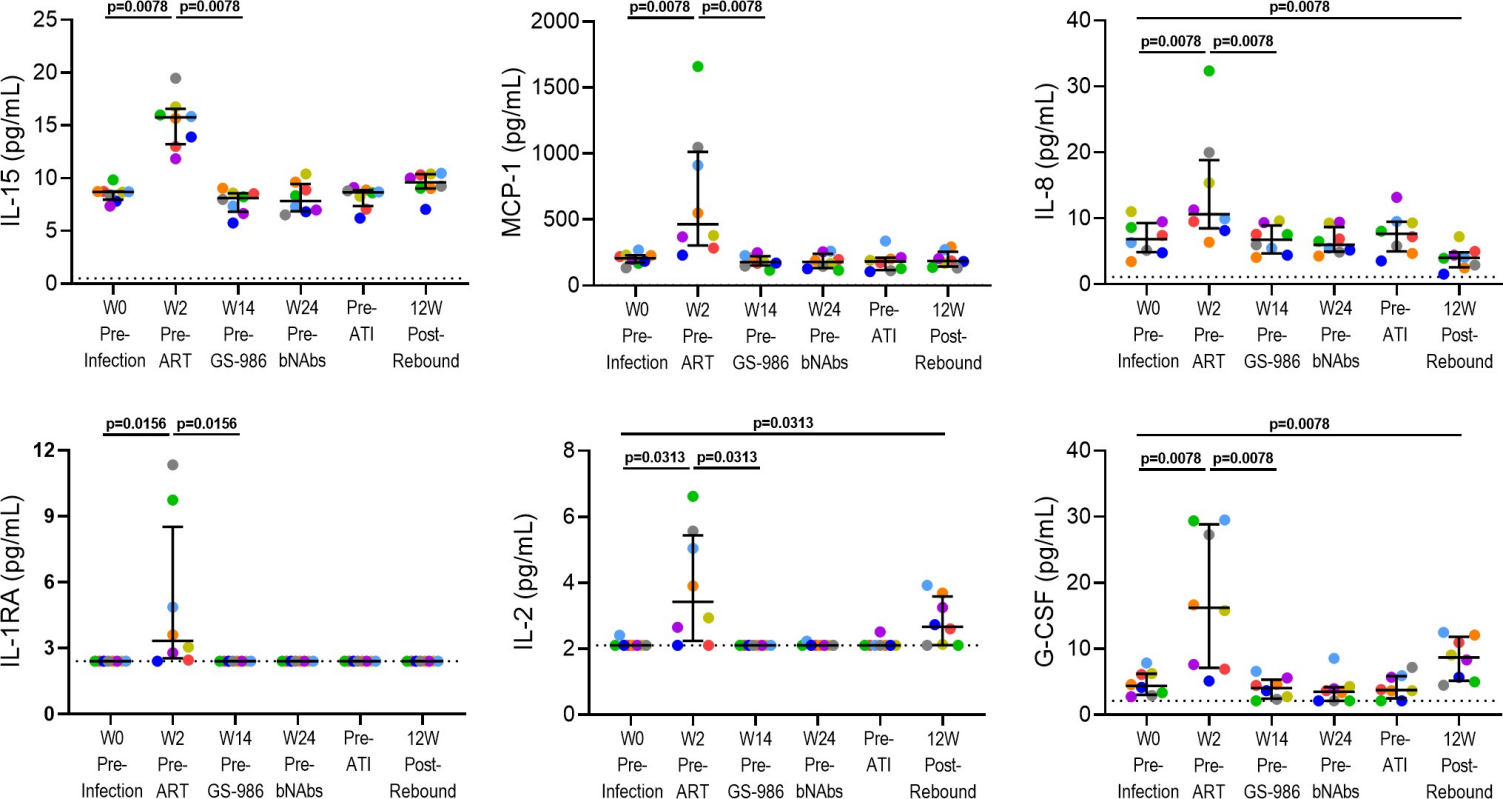
No evidence of persistent immune activation in the CSF associated with GS-986 or bnAbs. Biomarkers of immune activation and inflammation in the CSF were measured using multiplex assays. Cytokines that were increased after SHIV infection are shown. Data from animals in the active arm are displayed in color which each color corresponding to individual animals identified in Fig 4A. Lines and error bars represent median and interquartile range. Dotted lines represent limit of detection for respective markers measured.

Monoclonal antibodies are known to penetrate the CNS poorly due to the blood-brain barrier. Thus, we examined the potential for GS-986 to induce viral expression that is not targeted for elimination due to inadequate levels of bnAb. No increases in CSF markers of immune activation were seen at wk 24 (2 wks after the 5^th^ dose of GS-986) or immediately prior to ATI, when compared with pre-infection levels (Fig 8). Furthermore, CSF SHIV RNA was undetectable in all animals at all sampling time-points after ART initiation (Fig 3). At 12 wks post rebound, CSF SHIV RNA was only detectable at low levels in 1 active and 1 control animal. These data suggest that administration of GS-986, N6-LS and PGT121 had no measurable negative impact on immune activation or reservoir size in the CNS.

## Discussion

This study demonstrated that the combination of TLR7 agonist and dual bnAbs delayed viral rebound after ART interruption by 2-fold in rhesus macaques that initiated viral suppressive ART 14 days after SHIV-1157ipd3N4 inoculation. This approach may represent a potential strategy to target the HIV reservoir in HIV-infected individuals.

GS-986 administration was associated with the induction of plasma markers of immune activation, consistent with previous studies [6,11–14]. GS-986 has also been demonstrated to induce transient cellular immune activation in CD8+, NK, CD4+ cells and monocytes in multiple studies [6,11–14]. In this study, viral reactivation was not detectable in the plasma 24 hrs post GS-986 dosing or in the tissues, including lymph node, colon and CSF 2 wks after the 5^th^ as well as final administration of GS-986. These results are consistent with recent studies performed in both SHIV [6] or SIV-infected [11,13,14] macaques on ART or in HIV-infected individuals [15]. The absence of detectable plasma viral reactivation does not preclude transient viral reactivation in tissue sites outside of sampling time points.

The induction of Gag-specific T cells is of importance as these cells are essential in viral control [16–19] and are thought to be key to HIV remission [20,21]. The mechanisms underlying this could not be clearly delineated in this study. A potential postulation is that GS-986 administrations induced transient low-level viral expression, stimulating immune responses either directly or through binding to bnAbs and the generation of a vaccinal effect. These mechanisms may have contributed to the delayed viral rebound in the active arm in this study.

To avoid confounding anti-viral effects from bnAbs on time to viral rebound, ART was interrupted 4 wks after plasma levels of both N6-LS and PGT121 were <0.25 ug/mL. This resulted in variations in the timing of ART interruption between the animals. Though no statistically significant differences in duration of viral suppression or timing of ART interruption were identified, the variation in ART duration may pose as a limitation of this study.

Importantly, our data showed that the administration of a combination of TLR7 agonist and dual bnAbs to animals that initiated ART early in the course of SHIV infection was associated with a delay in viral rebound after ART interruption, corroborating findings by Borducchi *et al*. [6]. In this study, ART was initiated 14 days post SHIV inoculation. The MHRP RV217 prospective cohort involving seronegative high-risk individuals who underwent twice-weekly HIV-1 RNA testing estimated the eclipse phase (the time between HIV-1 infection and a diagnosable infection by nucleic acid testing) to be a week [22]. Thus ART initiation at day 14 more closely mirrors what is feasible in HIV-infected individuals, including our current clinical RV254 cohort in Thailand, where individuals are diagnosed and initiated on ART at the earliest stages of HIV infection [23]. This one week difference in ART initiation resulted in a 1.5 log_10_copies/mL increase in median pre-ART SHIV RNA levels: 5.7 log_10_copies/mL in this study vs 4.2 log_10_copies/mL when ART was initiated at 7 days post infection and pre-ART SHIV RNA level was identified as a correlate of time to viral rebound [6]. In addition, the timing of administration of TLR7 agonist was also different (12 wks after ART initiation in this study, before the viral reservoir reached a steady-state vs 95 wks). The earlier initiation and longer duration of ART in the Borducchi et al study likely translated to a smaller reservoir, with viral DNA being largely undetectable in PBMC (vs median of 323 copies/million PBMC in this study) at the time the interventions were administered [6]. The smaller reservoir may thus be more amenable to elimination, allowing for some NHP to achieve remission when initiating ART 7 days post infection.

The number of doses of bnAbs administered (ranging from 2–5) were limited by the development of ADA. The development of anti-N6-LS antibody is not uncommon based on previous NHP studies (Dr. John Mascola, personal communication) and may have been further exacerbated by the high dose (30 mg/kg) administered. We hypothesize that induction of anti-N6-LS antibody led to the development of cross reactive anti-PGT121 antibody, likely to the common Fc regions. The shorter duration of bnAb exposure may also have reduced the efficacy of the TLR7 agonist and dual bnAb strategy. In a recent study by Barouch et al., in macaques that initiated ART 1 year after SHIV-SF162P3 infection and were virologically suppressed for 2.5 years, the administration of 10 infusions of PGT121 or GS-9721 (Fc-modified version of PGT121) with TLR7 agonist prevented viral rebound in 41% (7 of 17) of animals following ART interruption [24]. Finally, intrinsic differences between SHIV-1157ipd3N4 and SHIV-SF162P3 may also have contributed to differences in impact on viral rebound between the 3 studies.

In summary, the administration of the combination of TLR7 agonist (GS-986) with N6-LS and PGT121 to macaques that initiated ART 14 days after SHIV-1157ipd3N4 infection was associated with a modest delay in viral rebound, despite limited dosing of bnAbs due to ADA. The evaluation of this strategy in humans, where the development of xenogeneic anti-drug antibodies will not be an issue, would allow the assessment of the impact of extended dosing.

## Material and methods

### Ethics statement

Animals were housed at the AAALAC International-accredited, Armed Forces Research Institute of Medical Science (AFRIMS; Bangkok, Thailand). The study was approved by the AFRIMS Institutional Animal Care and Use Committee under protocol number PN17-01. Research was conducted in compliance with Thai laws, the Animal Welfare Act and other U.S. federal statutes and regulations relating to animals and experiments involving animals and adheres to principles stated in the Guide for the Care and Use of Laboratory Animals, 2011 edition [25].

### Study design

Sixteen male, adult, Indian-origin rhesus macaque (*Macaca mulatta*), pre-screened to exclude protective MHC alleles (MamuA*01, B*08 and B*17), were inoculated intrarectally with SHIV-1157ipd3N4 (an R5-tropic, mucosally transmissible virus, constructed using SIVmac239 backbone, encoding an HIV subtype C env derived from a Zambian infant [26], at 3.9 x10e^7^ RNA copies, at week 0. Animals were randomly assigned into active or control arms whilst balancing Trim 5 alpha status, day 14 plasma SHIV RNA, weight and age. Daily ART (9-(2-phosphonomethoxypropyl) adenine, PMPA 20 mg/kg, emtricitabine 40 mg/kg and dolutegravir 2.5 mg/kg), was administered subcutaneously, from Day 14, as previously described [27]. Animals in the active arm (n = 8) received GS-986 (0.1 mg/kg) via oral gavage every 2 wks from wks 14 to 32 and intravenous N6-LS (30 mg/kg) and PGT121 (10 mg/kg) every 2 wks from wks 24 to 32, unless anti-drug antibodies (ADA) were detected. Animals were monitored for the development of ADA to N6-LS and PGT121 seven days after each dose. The administration of the respective bnAb was suspended after detection of ADA. ART was ceased when plasma levels of N6-LS and PGT121 were <0.25 ug/mL for 4 wks. Animals in the control arm (n = 8) received intravenous normal saline infusions at wk 24–32 and ceased ART at wk 40. During ART interruption, plasma SHIV RNA was assessed twice weekly to monitor for viral rebound. Animals were humanely euthanized 12 wks post viral rebound.

### Quantitation of SHIV RNA

Plasma, CSF and tissue SHIV-RNA levels were measured using real-time quantitative PCR as previously described [28,29]. Limit of quantification of the assay was 10 copies/mL.

### Quantitation of total SHIV DNA

Total cellular DNA was isolated from ~5x10^6^ PBMCs by proteinase K (Invitrogen, Carlsbad, California, USA) lysis. Viral DNA was measured by real-time qPCR in triplicate, and the number of copies was calculated based on parallel quantitation of a standard dilution of 3D8 cells containing a single copy of integrated SIV genomic DNA. qPCR was performed using TaqMan Fast Advanced Master Mix (Applied Biosystems, Vilnius, Lithuania), according to the manufacturer’s instructions, with SIV gag qPCR primer and probe sequences as previously described [30–32]. SIV *gag* copy numbers were normalized to rhesus albumin gene (*alb*) copy numbers detected by qPCR using a 10-fold dilution of PBMC from a healthy control rhesus macaque as the standard, as follows: (*gag* copies)/(*alb* copies) X 2(alb copies)/cell.

### Measurement of plasma bNAb levels

Plasma N6-LS and PGT121 concentrations were measured by ELISA as previously described [33]. Maxisorp ELISA plates (Thermo Fisher Scientific, Roskilde, Denmark) were coated with anti-idiotype Ab for N6-LS or PGT121 at 2 ug/mL, 100 uL/well, overnight at 4°C. Plates were then washed with PBS (Sigma Aldrich, St. Louis, Missouri, USA) –0.05% Tween 20 (Amresco, Solon, Ohio, USA) and blocked for 1 h at room temperature with Tris-Buffered Saline (TBS, Sigma Aldrich, St. Louis, Missouri, USA) with 5% nonfat dried milk powder (AppliChem, Damstadt, Germany) and 2% bovine serum albumin (BSA, Sigma Aldrich, St. Louis, Missouri, USA) and then washed. Standard curve calibrators (8 serial 2.5-fold dilutions of N6-LS or PGT121), spiked plasma control (10 ug/mL) and heat-inactivated plasma samples (3 serial 5-fold dilutions) were plated in duplicate and incubated for 1 h at room temperature. Plates were washed and incubated with donkey anti-human IgG (Fcγ-fragment specific)-HRP (Jackson Immunoresearch, Chester, Pennsylvania, USA) diluted to 1:10000 in blocking buffer for 30 mins at room temperature. Plates were washed again and developed with KPL SureBlue TMB Microwell Peroxidase Substrate (Seracare, Gaithersburg, Maryland, USA) for 20 mins followed by the addition of 1N Sulfuric acid (Fisher Chemical, Bergen County, New Jersey, USA). Plates were read at 450 nm on a SpectraMax microplate reader (Molecular Device, Sunnyvale, California, USA) using Softmax Pro version 4.3.1 software. The SoftMax Pro software calculated 4-Parameter curve fits for the standard calibrators and the test sample concentrations were determined by interpolation into the calibration curves.

### Measurement of lymph node and gut bNAb levels

Sigmoid colon and lymph node samples were thawed on ice, homogenized for 30 seconds in 200 μl elution buffer (PBS and EDTA-free complete protease inhibitor, Roche Diagnostics GmbH, Mannheim, Germany). The homogenate was cleared by centrifugation for 15 min at 4°C and the supernatant was filtered (0.22 μm; Corning, NY, USA). Levels of N6-LS and PGT121 antibodies were measured by ELISA, as above. The main difference was that tissue homogenate was diluted 2-fold with blocking buffer.

### Detection of ADA

Plasma anti-N6-LS and anti-PGT121 antibodies were measured by ELISA as previously described [34]. Maxisorp ELISA plates (Thermo Fisher Scientific, Roskilde, Denmark) were coated with N6-LS or PGT121 at 2 ug/mL, 100 uL/well, overnight at 4°C. Plates were then washed with PBS–0.05% Tween 20 and blocked for 1 h at room temperature with TBS with 5% nonfat dried milk power and 2% BSA and then washed. Heat-inactivated plasma samples (7 serial 5-fold dilutions) were plated in duplicate and incubated for 1 h at room temperature. Plates were washed. Wells were incubated with mouse anti-monkey IgG-HRP (Southern Biotechnology Associates, Birmingham, Alabama, USA) diluted to 1:8000 in blocking buffer for 30 mins at room temperature. Plates were washed again and developed with KPL SureBlue TMB Microwell Peroxidase Substrate for 20 mins followed by the addition of 1N Sulfuric acid. Plates were read at 450 nm on a SpectraMax microplate reader. The presence of ADA was defined to be >10-fold increase in end-point titer.

### Measurement of plasma cytokine levels

Plasma and CSF soluble markers of immune activation (including G-CSF, GM-CSF, IFNγ, IL-1β, IL-1ra, IL-2, IL-4, IL-5, IL-6, IL-8, IL-10, IL-12/23(p40), IL-13, IL-15, IL-17A, MCP-1, MIP-1β, MIP-1α, sD40L, TGF-α, TNF-α, VEGF, and IL-18) were quantified using the MILLIPLEX MAP Non-Human Primate Cytokine Magnetic Bead Panel (EMD Millipore Corporation, Billerica, Massachusetts, USA) as per manufacturer instructions, as previously described [29].

Plasma IFN-α was measured using the VeriKine Cynomolgus/Rhesus IFN-α ELISA Kit (PBL assay science, Piscataway, New Jersey, USA) as per manufacturer’s instructions.

### Intracellular cytokine staining (ICS)

Cryopreserved PBMCs were thawed, washed and resuspended in RPMI 1640 containing 10% human AB serum (HAB; Gemini Bio-Product, West Sacramento, CA, USA) and directly used for intracellular cytokine staining. One million PBMCs were stimulated for 6 hours in the presence of peptide pools representing HIV-1 Consensus C Env and SIVmac239 Gag (NIH AIDS Reagent Program, Germantown, MD, USA) at the final concentration of 1μg/peptide/ml or the positive control Phorbolmyristate acetate and Ionomycin (1μg/ml, Sigma-Aldrich, St. Louis, MO, USA). Included were the protein transport inhibitors monensin (BD Bioscience, San Jose, CA, USA), brefeldin A (BD Bioscience, San Jose, CA, USA), anti-CD49d co-stimulatory antibody and anti-CD107a BV785 (Biolegend, San Diego, CA, USA). DMSO stimulated wells, matching the highest concentration used in antigen wells were used as negative control. After stimulation, cells were washed and stained with Violet Live/Dead dye (Invitrogen, Eugene, Oregon, USA*)*. *Cells were then washed and* stained with anti-CCR7 BV711 (Biolegend, San Diego, CA, USA) for 20 min at 37°C, followed by surface staining with anti-CD45RA PE-Cy7 (BD Bioscience, San Jose, CA, USA), anti-CD4 BV510 (Biolegend, San Diego, CA, USA), anti-CD28 PE-Cy5 (BD Pharmingen, San Diego, CA, USA) and anti-CD69 ECD (Beckman Coulter, Brea, CA, USA) for 30 min at room temperature. Cells were fixed in 2% paraformaldehyde (Electron Microscopy Science, Hatfield, PA, USA), washed and incubated with 1X Perm/Wash (BD Bioscience, San Jose, CA, USA) for 15 min at room temperature and subsequently stained with anti-CD3 APC-Cy7 (BD Bioscience, San Jose, CA, USA), anti-CD8 BV605 (Biolegend, San Diego, CA, USA), anti-IFN-g APC (BD Pharmingen, San Diego, CA, USA), anti-IL-2 BV650 (BD Horizon, San Diego, CA, USA), anti-TNFa AlexaFluor700 (BD Pharmingen, San Diego, CA, USA), anti-IL-17A AlexaFluor488 (Biolegend, San Diego, CA, USA) and anti-Granzyme B PE (Invitrogen, Eugene, OR, USA*) for* 30 min at room temperature.

Cells were acquired within 24 hours using a custom built LSRFortessa flow cytometer (BD, San Jose, CA, USA) and analyzed using FlowJo software version 10.5.3 or higher (TreeStar, Ashland,OR, USA). A positive ICS response was defined as ≥0.05% gated positive cells after background control subtracted.

### Statistical analyses

Analysis of virologic and immunologic data was performed using GraphPad Prism v8.2.0 (GraphPad Software). Comparisons between different time-points in the same animals were performed using Wilcoxon matched-pairs signed rank test. Comparisons between animals in the 2 arms were performed using two-sided Mann–Whitney tests. Correlations were assessed by two-sided Spearman rank-correlation tests. Analysis of time to viral rebound was performed using Log-rank (Mentel-Cox) test.

## Supporting information

S1 Fig Timing of ART interruption and correlates of time to rebound. (A) ART interruption (ATI) occurred at wk 40 in animals in the control arm and at 4 wks after levels of both N6-LS and PGT121 became <0.25 ug/mL for animals in the active arm. (B) The duration of viral suppression on ART (SHIV RNA ≤ 10 copies/mL) were not significantly different between arms. No significant correlations between time to plasma viral rebound and (C) wk2 (pre-ART) plasma SHIV RNA or (D) SHIV DNA in PBMC at the time of ART interruption or the number of doses of (E) GS-986 or (F) PGT121 administered were found. Data from animals in the active arm are displayed in color which each color corresponding to individual animals identified in Fig 4A. Data from animals in the control arm are displayed as gray squares. Lines and error bars represent median and interquartile range.
(TIF)
