# Supplementary figures and images for: TLR7 agonist, N6-LS and PGT121 delayed viral rebound in SHIV-infected macaques after antiretroviral therapy interruption
Source: PLoS Pathog. 2021 Feb 18;17(2):e1009339. doi: 10.1371/journal.ppat.1009339 (PMC7924766; doi:10.1371/journal.ppat.1009339)

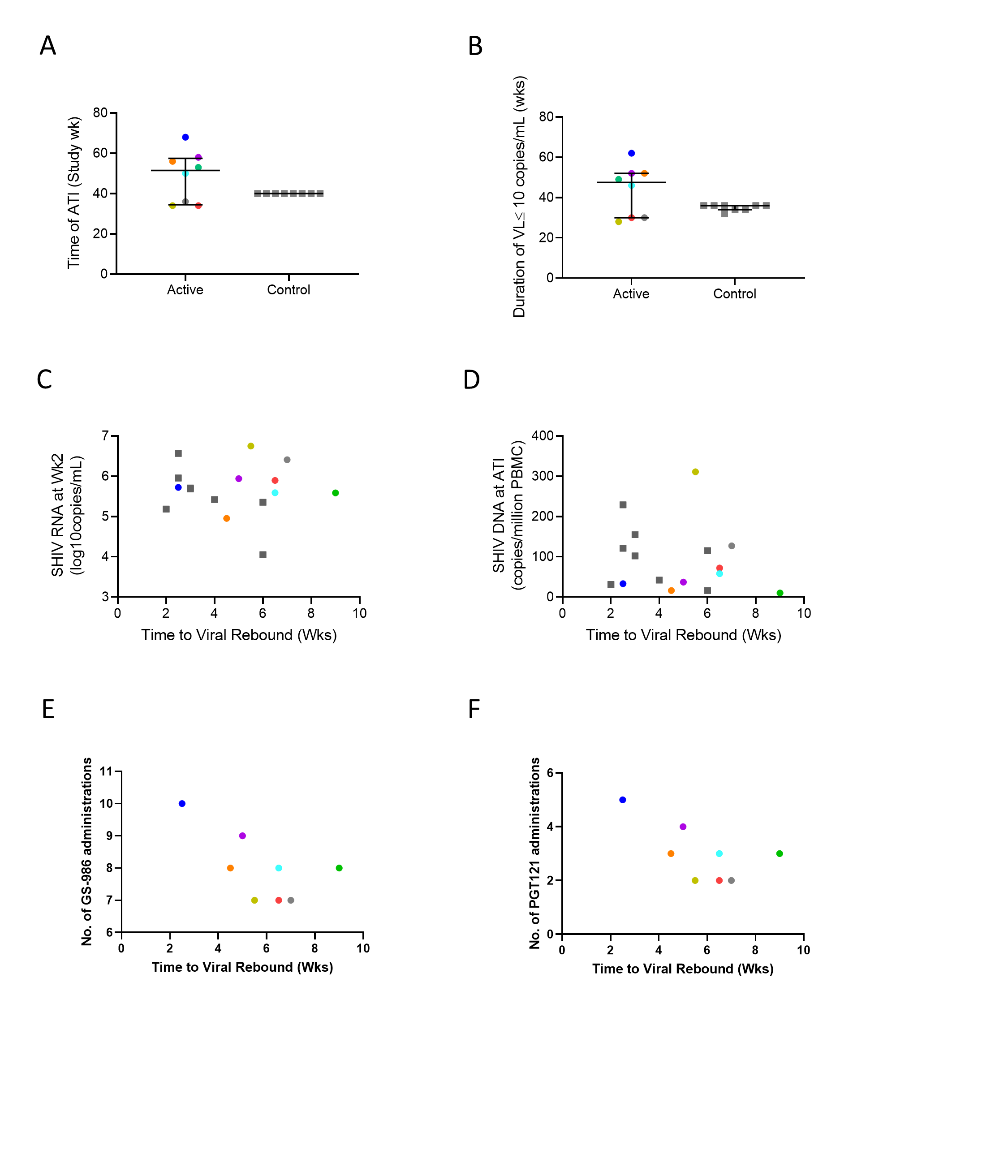

Supplement: S1 Fig — (A) ART interruption (ATI) occurred at wk 40 in animals in the control arm and at 4 wks after levels of both N6-LS and PGT121 became <0.25 ug/mL for animals in the active arm. (B) The duration of viral suppression on ART (SHIV RNA ≤ 10 copies/mL) were not significantly different between arms. No significant correlations between time to plasma viral rebound and (C) wk2 (pre-ART) plasma SHIV RNA or (D) SHIV DNA in PBMC at the time of ART interruption or the number of doses of (E) GS-986 or (F) PGT121 administered were found. Data from animals in the active arm are displayed in color which each color corresponding to individual animals identified in Fig 4A. Data from animals in the control arm are displayed as gray squares. Lines and error bars represent median and interquartile range. (TIF) [file ppat.1009339.s001.TIF]
